# Approximations of Shannon Mutual Information for Discrete Variables with Applications to Neural Population Coding

**DOI:** 10.3390/e21030243

**Published:** 2019-03-04

**Authors:** Wentao Huang, Kechen Zhang

**Affiliations:** 1 Key Laboratory of Cognition and Intelligence and Information Science Academy of China Electronics Technology Group Corporation, Beijing 100086, China; 2 Department of Biomedical Engineering, Johns Hopkins University School of Medicine, Baltimore, MD 21205, USA

**Keywords:** neural population coding, mutual information, Kullback-Leibler divergence, Rényi divergence, Chernoff divergence, approximation, discrete variables

## Abstract

Although Shannon mutual information has been widely used, its effective calculation is often difficult for many practical problems, including those in neural population coding. Asymptotic formulas based on Fisher information sometimes provide accurate approximations to the mutual information but this approach is restricted to continuous variables because the calculation of Fisher information requires derivatives with respect to the encoded variables. In this paper, we consider information-theoretic bounds and approximations of the mutual information based on Kullback-Leibler divergence and Rényi divergence. We propose several information metrics to approximate Shannon mutual information in the context of neural population coding. While our asymptotic formulas all work for discrete variables, one of them has consistent performance and high accuracy regardless of whether the encoded variables are discrete or continuous. We performed numerical simulations and confirmed that our approximation formulas were highly accurate for approximating the mutual information between the stimuli and the responses of a large neural population. These approximation formulas may potentially bring convenience to the applications of information theory to many practical and theoretical problems.

## 1. Introduction

Information theory is a powerful tool widely used in many disciplines, including, for example, neuroscience, machine learning, and communication technology [1,2,3,4,5,6,7]. As it is often notoriously difficult to effectively calculate Shannon mutual information in many practical applications [8], various approximation methods have been proposed to estimate the mutual information, such as those based on asymptotic expansion [9,10,11,12,13], *k*-nearest neighbor [14], and minimal spanning trees [15]. Recently, Safaai et al. proposed a copula method for estimation of mutual information, which can be nonparametric and potentially robust [16]. Another approach for estimating the mutual information is to simplify the calculations by approximations based on information-theoretic bounds, such as the Cramér–Rao lower bound [17] and the van Trees’ Bayesian Cramér–Rao bound [18].

In this paper, we focus on mutual information estimation based on asymptotic approximations [19,20,21,22,23,24]. For encoding of continuous variables, asymptotic relations between mutual information and Fisher information have been presented by several researchers [19,20,21,22]. Recently, Huang and Zhang [24] proposed an improved approximation formula, which remains accurate for high-dimensional variables. A significant advantage of this approach is that asymptotic approximations are sometimes very useful in analytical studies. For instance, asymptotic approximations allow us to prove that the optimal neural population distribution that maximizes the mutual information between stimulus and response can be solved by convex optimization [24]. Unfortunately this approach does not generalize to discrete variables since the calculation of Fisher information requires partial derivatives of the likelihood function with respect to the encoded variables. For encoding of discrete variables, Kang and Sompolinsky [23] represented an asymptotic relationship between mutual information and Chernoff information for statistically independent neurons in a large population. However, Chernoff information is still hard to calculate in many practical applications.

Discrete stimuli or variables occur naturally in sensory coding. While some stimuli are continuous (e.g., the direction of movement, and the pitch of a tone), others are discrete (e.g., the identities of faces, and the words in human speech). For definiteness, in this paper, we frame our questions in the context of neural population coding; that is, we assume that the stimuli or the input variables are encoded by the pattern of responses elicited from a large population of neurons. The concrete examples used in our numerical simulations were based on Poisson spike model, where the response of each neuron is taken as the spike count within a given time window. While this simple Poisson model allowed us to consider a large neural population, it only captured the spike rate but not any temporal structure of the spike trains [25,26,27,28]. Nonetheless, our mathematical results are quite general and should be applicable to other input–output systems under suitable conditions to be discussed later.

In the following, we first derive several upper and lower bounds on Shannon mutual information using Kullback-Leibler divergence and Rényi divergence. Next, we derive several new approximation formulas for Shannon mutual information in the limit of large population size. These formulas are more convenient to calculate than the mutual information in our examples. Finally, we confirm the validity of our approximation formulas using the true mutual information as evaluated by Monte Carlo simulations.

## 2. Theory and Methods

### 2.1. Notations and Definitions

Suppose the input x is a *K*-dimensional vector, $x={\left(x_{1},\cdots ,x_{K}\right)}^{T}$, which could be interpreted as the parameters that specifies a stimulus for a sensory system, and the outputs is an *N*-dimensional vector, $r={\left(r_{1},\cdots ,r_{N}\right)}^{T}$, which could be interpreted as the responses of *N* neurons. We assume *N* is large, generally N≫K. We denote random variables by upper case letters, e.g., random variables *X* and *R*, in contrast to their vector values x and r. The mutual information between *X* and *R* is defined by

(1) $$I=I\left(X;R\right)={\langle \ln\frac{p(r|x)}{p(r)}\rangle }_{r,x},$$

where $x\in X\subseteq R^{K}$, $r\in R\subseteq R^{N}$, and ${\langle \cdot \rangle }_{r,x}$ denotes the expectation with respect to the probability density function p(r,x). Similarly, in the following, we use ${\langle \cdot \rangle }_{r|x}$ and ${\langle \cdot \rangle }_{x}$ to denote expectations with respect to p(r|x) and p(x), respectively.

If p(x) and p(r|x) are twice continuously differentiable for almost every x∈X, then for large *N* we can use an asymptotic formula to approximate the true value of *I* with high accuracy [24]:

(2) $$I≃I_{G}=\frac{1}{2}{\langle \ln\left(\det\left(\frac{G(x)}{2\pi e}\right)\right)\rangle }_{x}+H\left(X\right),$$

which is sometimes reduced to

(3) $$I≃I_{F}=\frac{1}{2}{\langle \ln\left(\det\left(\frac{J(x)}{2\pi e}\right)\right)\rangle }_{x}+H\left(X\right),$$

where $\det\left(\cdot \right)$ denotes the matrix determinant, $H\left(X\right)=-{\langle \ln p(x)\rangle }_{x}$ is the stimulus entropy,

(4) $$\begin{array}{c} G\left(x\right)=J\left(x\right)+P\left(x\right), \end{array}$$

(5) $$\begin{array}{c} P\left(x\right)=-\frac{\partial^{2}\ln p\left(x\right)}{\partial x\partial x^{T}}, \end{array}$$

and

(6) $$J\left(x\right)=-{\langle \frac{\partial^{2}\ln p\left(r|x\right)}{\partial x\partial x^{T}}\rangle }_{r|x}={\langle \frac{\partial \ln p(r|x)}{\partial x}\frac{\partial \ln p(r|x)}{\partial x^{T}}\rangle }_{r|x}$$

is the Fisher information matrix.

We denote the Kullback-Leibler divergence as

(7) $$D\left(x||\hat{x}\right)={\langle \ln\frac{p\left(r|x\right)}{p\left(r|\hat{x}\right)}\rangle }_{r|x},$$

and denote Rényi divergence [29] of order β+1 as

(8) $$D_{\beta }\left(x||\hat{x}\right)=\frac{1}{\beta }\ln{\langle {\left(\frac{p(r|x)}{p\left(r|\hat{x}\right)}\right)}^{\beta }\rangle }_{r|x}.$$

Here, $\beta D_{\beta }\left(x||\hat{x}\right)$ is equivalent to Chernoff divergence of order β+1 [30]. It is well known that $D_{\beta }\left(x||\hat{x}\right)\to D\left(x||\hat{x}\right)$ in the limit β→0.

We define

(9) $$\begin{array}{cc} I_{u} & =-{\langle \ln{\langle \exp\left(-D\left(x||\hat{x}\right)\right)\rangle }_{\hat{x}}\rangle }_{x}, \end{array}$$

(10) $$\begin{array}{cc} I_{e} & =-{\langle \ln{\langle \exp\left(-e^{-1}D\left(x||\hat{x}\right)\right)\rangle }_{\hat{x}}\rangle }_{x}, \end{array}$$

(11) $$\begin{array}{cc} I_{\beta ,\alpha } & =-{\langle \ln{\langle \exp\left(-\beta D_{\beta }\left(x||\hat{x}\right)+\left(1-\alpha \right)\ln\frac{p\left(x\right)}{p\left(\hat{x}\right)}\right)\rangle }_{\hat{x}}\rangle }_{x}, \end{array}$$

where in $I_{\beta ,\alpha }$ we have $\beta \in \left(0,\;1\right)$ and $\alpha \in \left(0,\;\infty \right)$ and assume $p\left(x\right)>0$ for all x∈X.

In the following, we suppose x takes *M* discrete values, $x_{m}$, $m\in M=\left\{1,\;2,\;\cdots ,\;M\right\}$, and $p(x_{m})>0$ for all *m*. Now, the definitions in Equations (9)–(11) become

(12) $$\begin{array}{cc} I_{u} & =-\sum_{m=1}^{M}p\left(x_{m}\right)\ln\left(\sum_{\hat{m}=1}^{M}\frac{p\left(x_{\hat{m}}\right)}{p\left(x_{m}\right)}\exp\left(-D\left(x_{m}\left|\right|x_{\hat{m}}\right)\right)\right)+H\left(X\right), \end{array}$$

(13) $$\begin{array}{cc} I_{e} & =-\sum_{m=1}^{M}p\left(x_{m}\right)\ln\left(\sum_{\hat{m}=1}^{M}\frac{p\left(x_{\hat{m}}\right)}{p\left(x_{m}\right)}\exp\left(-e^{-1}D\left(x_{m}\left|\right|x_{\hat{m}}\right)\right)\right)+H\left(X\right), \end{array}$$

(14) $$\begin{array}{cc} I_{\beta ,\alpha } & =-\sum_{m=1}^{M}p\left(x_{m}\right)\ln\left(\sum_{\hat{m}=1}^{M}{\left(\frac{p(x_{\hat{m}})}{p(x_{m})}\right)}^{\alpha }\exp\left(-\beta D_{\beta }\left(x_{m}\left|\right|x_{\hat{m}}\right)\right)\right)+H\left(X\right). \end{array}$$

Furthermore, we define

(15) $$\begin{array}{cc} I_{d} & =-\sum_{m=1}^{M}p\left(x_{m}\right)\ln\left(1+\sum_{\hat{m}\in M_{m}^{u}}\frac{p(x_{\hat{m}})}{p(x_{m})}\exp\left(-e^{-1}D\left(x_{m}\left|\right|x_{\hat{m}}\right)\right)\right)+H\left(X\right), \end{array}$$

(16) $$\begin{array}{cc} I_{u}^{d} & =-\sum_{m=1}^{M}p\left(x_{m}\right)\ln\left(1+\sum_{\hat{m}\in M_{m}^{u}}\frac{p(x_{\hat{m}})}{p(x_{m})}\exp\left(-D\left(x_{m}\left|\right|x_{\hat{m}}\right)\right)\right)+H\left(X\right), \end{array}$$

(17) $$\begin{array}{cc} I_{\beta ,\alpha }^{d} & =-\sum_{m=1}^{M}p\left(x_{m}\right)\ln\left(1+\sum_{\hat{m}\in M_{m}^{\beta }}{\left(\frac{p(x_{\hat{m}})}{p(x_{m})}\right)}^{\alpha }\exp\left(-\beta D_{\beta }\left(x_{m}\left|\right|x_{\hat{m}}\right)\right)\right)+H\left(X\right), \end{array}$$

(18) $$\begin{array}{cc} I_{D} & =-\sum_{m=1}^{M}p\left(x_{m}\right)\ln\left(1+\sum_{\hat{m}\in M_{m}^{u}}\exp\left(-e^{-1}D\left(x_{m}\left|\right|x_{\hat{m}}\right)\right)\right)+H\left(X\right), \end{array}$$

where

(19) $$\begin{array}{c} {\overset{ˇ}{M}}_{m}^{\beta }=\left\{\hat{m}:\hat{m}=\underset{\overset{ˇ}{m}\in M-{\hat{M}}_{m}^{\beta }}{\arg\;\min\;}D_{\beta }\left(x_{m}\left|\right|x_{\overset{ˇ}{m}}\right)\right\}, \end{array}$$

(20) $$\begin{array}{c} {\overset{ˇ}{M}}_{m}^{u}=\left\{\hat{m}:\hat{m}=\underset{\overset{ˇ}{m}\in M-{\hat{M}}_{m}^{u}}{\arg\;\min\;}D\left(x_{m}\left|\right|x_{\overset{ˇ}{m}}\right)\right\}, \end{array}$$

(21) $$\begin{array}{c} {\hat{M}}_{m}^{\beta }=\left\{\hat{m}:D_{\beta }\left(x_{m}\left|\right|x_{\hat{m}}\right)=0\right\}, \end{array}$$

(22) $$\begin{array}{c} {\hat{M}}_{m}^{u}=\left\{\hat{m}:D\left(x_{m}\left|\right|x_{\hat{m}}\right)=0\right\}, \end{array}$$

(23) $$\begin{array}{c} M_{m}^{\beta }={\overset{ˇ}{M}}_{m}^{\beta }\cup {\hat{M}}_{m}^{\beta }-\left\{m\right\}, \end{array}$$

(24) $$\begin{array}{c} M_{m}^{u}={\overset{ˇ}{M}}_{m}^{u}\cup {\hat{M}}_{m}^{u}-\left\{m\right\}. \end{array}$$

Here, notice that, if x is uniformly distributed, then by definition $I_{d}$ and $I_{D}$ become identical. The elements in set ${\overset{ˇ}{M}}_{m}^{\beta }$ are those that make $D_{\beta }\left(x_{m}\left|\right|x_{\overset{ˇ}{m}}\right)$ take the minimum value, excluding any element that satisfies the condition $D_{\beta }\left(x_{m}\left|\right|x_{\hat{m}}\right)=0$. Similarly, the elements in set ${\overset{ˇ}{M}}_{m}^{u}$ are those that minimize $D\left(x_{m}\left|\right|x_{\overset{ˇ}{m}}\right)$ excluding the ones that satisfy the condition $D\left(x_{m}\left|\right|x_{\hat{m}}\right)=0$.

### 2.2. Theorems

In the following, we state several conclusions as theorems and prove them in Appendix A.

**Theorem** **1.** *The mutual information I is bounded as follows:*

(25) $$I_{\beta ,\alpha }\leq I\leq I_{u}.$$

**Theorem** **2.** *The following inequalities are satisfied,*

(26) $$I_{\beta_{1},1}\leq I_{e}\leq I_{u}$$

*where $I_{\beta_{1},1}$ is a special case of $I_{\beta ,\alpha }$ in Equation (11) with $\beta_{1}=e^{-1}$ so that*

(27) $$I_{\beta_{1},1}=-{\langle \ln{\langle \exp\left(-\beta_{1}D_{\beta_{1}}\left(x||\hat{x}\right)\right)\rangle }_{\hat{x}}\rangle }_{x}.$$

**Theorem** **3.** *If there exist $\gamma_{1}>0$ and $\gamma_{2}>0$ such that*

(28) $$\begin{array}{cc} \beta D_{\beta }\left(x_{m}\left|\right|x_{m_{1}}\right) & \geq \gamma_{1}\ln N, \end{array}$$

(29) $$\begin{array}{cc} D\left(x_{m}\left|\right|x_{m_{2}}\right) & \geq \gamma_{2}\ln N, \end{array}$$

*for discrete stimuli $x_{m}$, where m∈M, $m_{1}\in M-M_{m}^{\beta }$ and $m_{2}\in M-M_{m}^{u}$, then we have the following asymptotic relationships:*

(30) $$I_{\beta ,\alpha }=I_{\beta ,\alpha }^{d}+O\left(N^{-\gamma_{1}}\right)\leq I\leq I_{u}=I_{u}^{d}+O\left(N^{-\gamma_{2}}\right)$$

*and*

(31) $$I_{e}=I_{d}+O\left(N^{-\gamma_{2}/e}\right).$$

**Theorem** **4.** *Suppose p(x) and p(r|x) are twice continuously differentiable for x∈X, $∥q{}^{\prime }\left(x\right)∥<\infty$, $∥q^{\prime \prime }\left(x\right)∥<\infty$, where q(x)=lnp(x) and ${}^{\prime }$ and ${}^{\prime \prime }$ denote partial derivatives ∂/∂x and $\partial^{2}/\partial x\partial x^{T}$, and $G_{\gamma }\left(x\right)$ is positive definite with $∥NG_{\gamma }^{-1}\left(x\right)∥=O\left(1\right)$, where $∥\cdot ∥$ denotes matrix Frobenius norm,*

(32) $$G_{\gamma }\left(x\right)=\gamma \left(J\left(x\right)+P\left(x\right)\right),$$

*$\gamma =\beta \left(1-\beta \right)$ and $\beta \in \left(0,\;1\right)$. If there exist an $\omega =\omega \left(x\right)>0$ such that*

(33) $$\begin{array}{c} \det{\left(G(x)\right)}^{1/2}\int_{{\bar{X}}_{\varepsilon }\left(x\right)}p\left(\hat{x}\right)\exp\left(-D\left(x||\hat{x}\right)\right)d\hat{x}=O\left(N^{-1}\right), \end{array}$$

(34) $$\begin{array}{c} \det{\left(G_{\gamma }\left(x\right)\right)}^{1/2}\int_{{\bar{X}}_{\varepsilon }\left(x\right)}p\left(\hat{x}\right)\exp\left(-\beta D_{\beta }\left(x||\hat{x}\right)\right)d\hat{x}=O\left(N^{-1}\right), \end{array}$$

*for all x∈X and $\varepsilon \in \left(0,\;\omega \right)$, where ${\bar{X}}_{\omega }\left(x\right)=X-X_{\omega }\left(x\right)$ is the complementary set of $X_{\omega }\left(x\right)=\left\{\overset{˘}{x}\in R^{K}:{\left(\overset{˘}{x}-x\right)}^{T}G\left(x\right)\left(\overset{˘}{x}-x\right)<N\omega^{2}\right\}$, then we have the following asymptotic relationships:*

(35) $$I_{\beta ,\alpha }\leq I_{\gamma_{0}}+O\left(N^{-1}\right)\leq I\leq I_{u}=I_{G}+K/2+O\left(N^{-1}\right),$$

(36) $$I_{e}=I_{G}+O\left(N^{-1}\right),$$

(37) $$I_{\beta ,\alpha }=I_{\gamma }+O\left(N^{-1}\right),$$

*where*

(38) $$I_{\gamma }=\frac{1}{2}\int_{X}p\left(x\right)\ln\left(\det\left(\frac{G_{\gamma }\left(x\right)}{2\pi }\right)\right)dx+H\left(X\right)$$

*and $\gamma_{0}=\beta_{0}\left(1-\beta_{0}\right)=1/4$ with $\beta_{0}=1/2$.*

**Remark** **1.** *We see from Theorems 1 and 2 that the true mutual information I and the approximation $I_{e}$ both lie between $I_{\beta_{1},1}$ and $I_{u}$, which implies that their values may be close to each other. For discrete variable x, Theorem 3 tells us that $I{}_{e}$ and $I_{d}$ are asymptotically equivalent (i.e., their difference vanishes) in the limit of large N. For continuous variable x, Theorem 4 tells us that $I{}_{e}$ and $I_{G}$ are asymptotically equivalent in the limit of large N, which means that $I_{e}$ and I are also asymptotically equivalent because $I_{G}$ and I are known to be asymptotically equivalent [24].*

**Remark** **2.** *To see how the condition in Equation (33) could be satisfied, consider the case where $D\left(x||\hat{x}\right)$ has only one extreme point at $\hat{x}=x$ for $\hat{x}\in X_{\omega }\left(x\right)$ and there exists an η>0 such that $N^{-1}D\left(x|\hat{x}\right)\geq \eta$ for $\hat{x}\in {\bar{X}}_{\omega }\left(x\right)$. Now, the condition in Equation (33) is satisfied because*

(39) $$\begin{array}{c} \det{\left(G(x)\right)}^{1/2}\int_{{\bar{X}}_{\varepsilon }\left(x\right)}p\left(\hat{x}\right)\exp\left(-D\left(x||\hat{x}\right)\right)d\hat{x}\\ \leq \det{\left(G(x)\right)}^{1/2}\int_{{\bar{X}}_{\varepsilon }\left(x\right)}p\left(\hat{x}\right)\exp\left(-\hat{\eta }\left(\varepsilon \right)N\right)d\hat{x}\\ =O\left(N^{K/2}e^{-\hat{\eta }\left(\varepsilon \right)N}\right), \end{array}$$

*where by assumption we can find an $\hat{\eta }\left(\varepsilon \right)>0$ for any given $\varepsilon \in \left(0,\;\omega \right)$. The condition in Equation (34) can be satisfied in a similar way. When $\beta_{0}=1/2$, $\beta_{0}D_{\beta_{0}}\left(x||\hat{x}\right)$ is the Bhattacharyya distance [31]:*

(40) $$\beta_{0}D_{\beta_{0}}\left(x||\hat{x}\right)=-\ln\left(\int_{R}\sqrt{p\left(r|x\right)p\left(r|\hat{x}\right)}dr\right),$$

*and we have*

(41) $$J\left(x\right)={\left.\frac{\partial^{2}\left(D\left(x||\hat{x}\right)\right)}{\partial \hat{x}\partial {\hat{x}}^{T}}\right|}_{\hat{x}=x}={\left.\frac{\partial^{2}\left(4\beta_{0}D_{\beta_{0}}\left(x||\hat{x}\right)\right)}{\partial \hat{x}\partial {\hat{x}}^{T}}\right|}_{\hat{x}=x}={\left.\frac{\partial^{2}\left(8H_{l}^{2}\left(x||\hat{x}\right)\right)}{\partial \hat{x}\partial {\hat{x}}^{T}}\right|}_{\hat{x}=x},$$

*where $H_{l}\left(x||\hat{x}\right)$ is the Hellinger distance [32] between p(r|x) and $p(r|\hat{x})$:*

(42) $$H_{l}^{2}\left(x||\hat{x}\right)=\frac{1}{2}\int_{R}{\left(\sqrt{p(r|x)}-\sqrt{p(r|\hat{x})}\right)}^{2}dr.$$

*By Jensen’s inequality, for $\beta \in \left(0,\;1\right)$ we get*

(43) $$0\leq D_{\beta }\left(x||\hat{x}\right)\leq D\left(x||\hat{x}\right).$$

*Denoting the Chernoff information [8] as*

(44) $$C\left(x||\hat{x}\right)=\max_{\beta \in \left(0,\;1\right)}\left(\beta D_{\beta }\left(x||\hat{x}\right)\right)=\beta_{m}D_{\beta_{m}}\left(x||\hat{x}\right),$$

*where $\beta D_{\beta }\left(x||\hat{x}\right)$ achieves its maximum at $\beta_{m}$, we have*

(45) $$\begin{array}{c} I_{\beta ,\alpha }-H\left(X\right)\\ \leq h_{c}=-\sum_{m=1}^{M}p\left(x_{m}\right)\ln\left(\sum_{\hat{m}=1}^{M}\frac{p\left(x_{\hat{m}}\right)}{p\left(x_{m}\right)}\exp\left(-C\left(x_{m}\left|\right|x_{\hat{m}}\right)\right)\right) \end{array}$$

(46) $$\begin{array}{c} \leq h_{d}=-\sum_{m=1}^{M}p\left(x_{m}\right)\ln\left(\sum_{\hat{m}=1}^{M}\frac{p\left(x_{\hat{m}}\right)}{p\left(x_{m}\right)}\exp\left(-\beta_{m}D_{\beta }\left(x_{m}\left|\right|x_{\hat{m}}\right)\right)\right). \end{array}$$

*By Theorem 4,*

(47) $$\begin{array}{c} \max_{\beta \in \left(0,\;1\right)}I_{\beta ,\alpha }=I_{\gamma_{0}}+O\left(N^{-1}\right), \end{array}$$

(48) $$\begin{array}{c} I_{\gamma_{0}}=I_{G}-\frac{K}{2}\ln\frac{4}{e}. \end{array}$$

*If $\beta_{m}=1/2$, then, by Equations (46)–(48) and (50), we have*

(49) $$\begin{array}{cc} \max_{\beta \in \left(0,\;1\right)}I_{\beta }+\frac{K}{2}\ln\frac{4}{e}+O\left(N^{-1}\right) & \leq I_{e}=I+O\left(N^{-1}\right)\\ & \leq h_{d}+H\left(X\right)\leq I_{u}. \end{array}$$

*Therefore, from Equations (45), (46) and (49), we can see that $I_{e}$ and I are close to $h_{c}+H\left(X\right)$.*

### 2.3. Approximations for Mutual Information

In this section, we use the relationships described above to find effective approximations to true mutual information *I* in the case of large but finite *N*. First, Theorems 1 and 2 tell us that the true mutual information *I* and its approximation $I_{e}$ lie between lower and upper bounds given by: $I_{\beta ,\alpha }\leq I\leq I_{u}$ and $I_{\beta_{1},1}\leq I_{e}\leq I_{u}$. As a special case, *I* is also bounded by $I_{\beta_{1},1}\leq I\leq I_{u}$. Furthermore, from Equations (2) and (36) we can obtain the following asymptotic equality under suitable conditions:

(50) $$I=I_{e}+O\left(N^{-1}\right).$$

Hence, for continuous stimuli, we have the following approximate relationship for large *N*:

(51) $$I≃I_{e}≃I_{G}.$$

For discrete stimuli, by Equation (31) for large but finite *N*, we have

(52) $$I≃I_{e}≃I_{d}=-\sum_{m=1}^{M}p\left(x_{m}\right)\ln\left(1+\sum_{\hat{m}\in M_{m}^{u}}\frac{p(x_{\hat{m}})}{p(x_{m})}\exp\left(-e^{-1}D\left(x_{m}\left|\right|x_{\hat{m}}\right)\right)\right)+H\left(X\right).$$

Consider the special case $p\left(x_{\hat{m}}\right)≃p\left(x_{m}\right)$ for $\hat{m}\in M_{m}^{u}$. With the help of Equation (18), substitution of $p\left(x_{\hat{m}}\right)≃p\left(x_{m}\right)$ into Equation (52) yields

(53) $$\begin{array}{cc} I & ≃I_{D}=-\sum_{m=1}^{M}p\left(x_{m}\right)\ln\left(1+\sum_{\hat{m}\in M_{m}^{u}}\exp\left(-e^{-1}D\left(x_{m}\left|\right|x_{\hat{m}}\right)\right)\right)+H\left(X\right)\\ & ≃-\sum_{m=1}^{M}p\left(x_{m}\right)\sum_{\hat{m}\in M_{m}^{u}}\exp\left(-e^{-1}D\left(x_{m}\left|\right|x_{\hat{m}}\right)\right)+H\left(X\right)\\ & =I_{D}^{0} \end{array}$$

where $I_{D}^{0}\leq I_{D}$ and the second approximation follows from the first-order Taylor expansion assuming that the term $\sum_{\hat{m}\in M_{m}^{u}}\exp\left(-e^{-1}D\left(x_{m}\left|\right|x_{\hat{m}}\right)\right)$ is sufficiently small.

The theoretical discussion above suggests that $I_{e}$ and $I_{d}$ are effective approximations to true mutual information *I* in the limit of large *N*. Moreover, we find that they are often good approximations of mutual information *I* even for relatively small *N*, as illustrated in the following section.

## 3. Results of Numerical Simulations

Consider Poisson model neuron whose responses (i.e., numbers of spikes within a given time window) follow a Poisson distribution [24]. The mean response of neuron *n*, with $n\in \left\{1,\;2,\;\cdots ,\;N\right\}$, is described by the tuning function $f\left(x;\;\theta_{n}\right)$, which takes the form of a Heaviside step function:

(54) $$f\left(x;\;\theta_{n}\right)=\left\{\begin{array}{cc} A, & \mathrm{if}\;x\geq \theta_{n},\\ 0, & \mathrm{if}\;x<\theta_{n}, \end{array}\right.$$

where the stimulus $x\in \left[-T,\;T\right]$ with T=10, A=10, and the centers $\theta_{1}$, $\theta_{2}$, ⋯, $\theta_{N}$ of the *N* neurons are uniformly spaced in interval $\left[-T,\;T\right]$, namely, $\theta_{n}=\left(n-1\right)d-T$ with d=2T/(N−1) for N≥2, and $\theta_{n}=0$ for N=1. We suppose that the discrete stimulus *x* has M=21 possible values that are evenly spaced from −T to *T*, namely, $x\in X=\left\{x_{m}:x_{m}=2\left(m-1\right)T/\left(M-1\right)-T,m=1,2,\cdots ,M\right\}$. Now, the Kullback-Leibler divergence can be written as

(55) $$D\left(x_{m}\left|\right|x_{\hat{m}}\right)=f\left(x_{m};\;\theta_{n}\right)\log\left(\frac{f\left(x_{m};\;\theta_{n}\right)}{f\left(x_{\hat{m}};\;\theta_{n}\right)}\right)+f\left(x_{\hat{m}};\;\theta_{n}\right)-f\left(x_{m};\;\theta_{n}\right).$$

Thus, we have $\exp\left(-e^{-1}D\left(x_{m}\left|\right|x_{\hat{m}}\right)\right)=1$ when $f\left(x_{m};\;\theta_{n}\right)=f\left(x_{\hat{m}};\;\theta_{n}\right)$, $\exp\left(-e^{-1}D\left(x_{m}\left|\right|x_{\hat{m}}\right)\right)=\exp\left(-e^{-1}A\right)$ when $f\left(x_{m};\;\theta_{n}\right)=0$ and $f\left(x_{\hat{m}};\;\theta_{n}\right)=A$, and $\exp\left(-e^{-1}D\left(x_{m}\left|\right|x_{\hat{m}}\right)\right)=0$ when $f\left(x_{m};\;\theta_{n}\right)=A$ and $f\left(x_{\hat{m}};\;\theta_{n}\right)=0$. Therefore, in this case, we have

(56) $$I_{e}=I_{d}.$$

More generally, this equality holds true whenever the tuning function has binary values.

**Figure 1:**
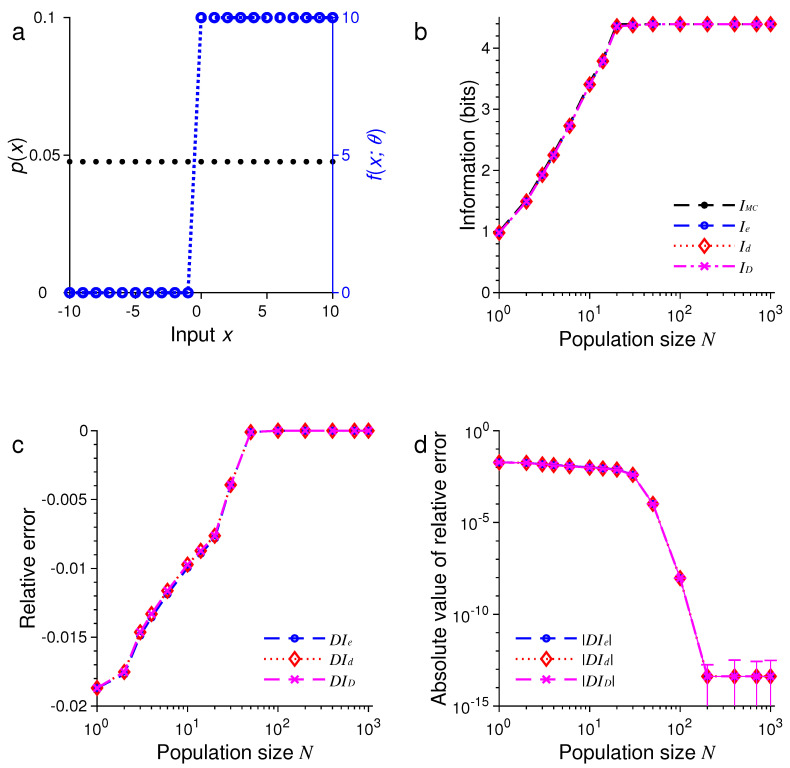
A comparison of approximations $I_{e}$, $I_{d}$ and $I_{D}$ against $I_{MC}$ obtained by Monte Carlo method for one-dimensional discrete stimuli. (**a**) Discrete uniform distribution of the stimulus p(x) (black dots) and the Heaviside step tuning function $f\left(x;\;\theta \right)$ with center θ=0 (blue dashed lines); (**b**) The values of $I_{MC}$, $I_{e}$, $I_{d}$ and $I_{D}$ depend on the population size or total number of neurons *N*; (**c**) The relative errors $DI_{e}$, $DI_{d}$ and $DI_{D}$ for the results in (**b**); (**d**) The absolute values of the relative errors $|DI_{e}|$, $|DI_{d}|$ and $|DI_{D}|$ as in (**c**), with error bars showing standard deviations of repeated trials.

In the first example, as illustrated in Figure 1, we suppose the stimulus has a uniform distribution, so that the probability is given by $p(x_{m})=1/M$. Figure 1a shows graphs of the input distribution p(x) and a representative tuning function $f\left(x;\;\theta \right)$ with the center θ=0.

To assess the accuracy of the approximation formulas, we employed Monte Carlo (MC) simulation to evaluate the mutual information *I* [24]. In our MC simulation, we first sampled an input $x_{j}\in X$ from the uniform distribution $p(x_{j})=1/M$, then generated the neural responses $r_{j}$ by the conditional distribution $p(r_{j}|x_{j})$ based on the Poisson model, where j=1, 2, ⋯, $j_{\max}$. The value of mutual information by MC simulation was calculated by

(57) $$I_{MC}^{*}=\frac{1}{j_{\max}}\sum_{j=1}^{j_{\max}}\ln\left(\frac{p(r_{j}|x_{j})}{p(r_{j})}\right),$$

where

(58) $$p\left(r_{j}\right)=\sum_{m=1}^{M}p\left(r_{j}|x_{m}\right)p\left(x_{m}\right).$$

To assess the precision of our MC simulation, we computed the standard deviation of repeated trials by bootstrapping:

(59) $$I_{std}=\sqrt{\frac{1}{i_{\max}}\sum_{i=1}^{i_{\max}}{\left(I_{MC}^{i}-I_{MC}\right)}^{2}},$$

where

(60) $$\begin{array}{cc} I_{MC}^{i} & =\frac{1}{j_{\max}}\sum_{j=1}^{j_{\max}}\ln\left(\frac{p(r_{\Gamma_{j,i}}|x_{\Gamma_{j,i}})}{p(r_{\Gamma_{j,i}})}\right), \end{array}$$

(61) $$\begin{array}{cc} I_{MC} & =\frac{1}{i_{\max}}\sum_{i=1}^{i_{\max}}I_{MC}^{i}, \end{array}$$

and $\Gamma_{j,i}\in \left\{1,\;2,\;\cdots ,\;j_{\max}\right\}$ is the $\left(j,i\right)$-th entry of the matrix $\Gamma \in N^{j_{\max}\times i_{\max}}$ with samples taken randomly from the integer set {1, 2, ⋯, $j_{\max}\}$ by a uniform distribution. Here, we set $j_{\max}=5\times 10^{5}$, $i_{\max}=100$ and $M=10^{3}$.

For different N∈{1,2,3,4,6,10,14,20,30,50,100,200,400,700,1000}, we compared $I_{MC}$ with $I_{e}$, $I_{d}$ and $I_{D}$, as illustrated in Figure 1b–d. Here, we define the relative error of approximation, e.g., for $I_{e}$, as

(62) $$DI_{e}=\frac{I_{e}-I_{MC}}{I_{MC}},$$

and the relative standard deviation

(63) $$DI_{std}=\frac{I_{std}}{I_{MC}}.$$

**Figure 2:**
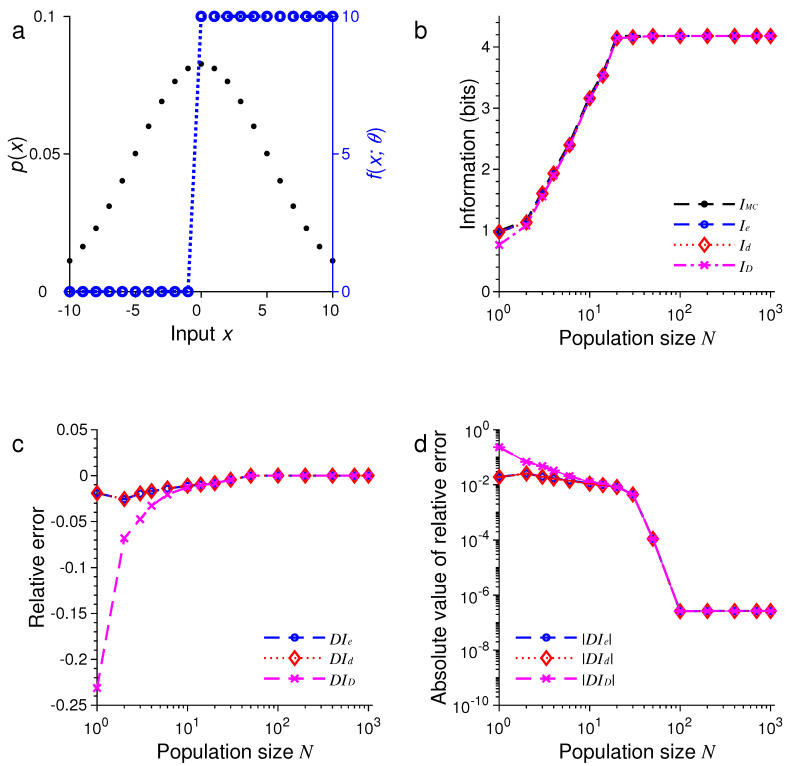
A comparison of approximations $I_{e}$, $I_{d}$ and $I_{D}$ against $I_{MC}$. The situation is identical to that in Figure 1 except that the stimulus distribution p(x) is peaked rather flat (black dots in (**a**)). (**a**) Discrete Gaussian-like distribution of the stimulus p(x) (black dots) and the Heaviside step tuning function $f\left(x;\;\theta \right)$ with center θ=0 (blue dashed lines); (**b**) The values of $I_{MC}$, $I_{e}$, $I_{d}$ and $I_{D}$ depend on the population size or total number of neurons *N*; (**c**) The relative errors $DI_{e}$, $DI_{d}$ and $DI_{D}$ for the results in (**b**); (**d**) The absolute values of the relative errors $|DI_{e}|$, $|DI_{d}|$ and $|DI_{D}|$ as in (**c**), with error bars showing standard deviations of repeated trials.

For the second example, we only changed the probability distribution of stimulus $p\left(x_{m}\right)$ while keeping all other conditions unchanged. Now, $p\left(x_{m}\right)$ is a discrete sample from a Gaussian function:

(64) $$p\left(x_{m}\right)=Z^{-1}\exp\left(-\frac{x_{m}^{2}}{2\sigma^{2}}\right),\;m=1,2,\cdots ,M,$$

where Z is the normalization constant and σ=T/2. The results are illustrated in Figure 2.

Next, we changed each tuning function $f\left(x;\;\theta_{n}\right)$ to a rectified linear function:

(65) $$f\left(x;\theta_{n}\right)=\max\left(0,x-\theta_{n}\right),$$

**Figure 3:**
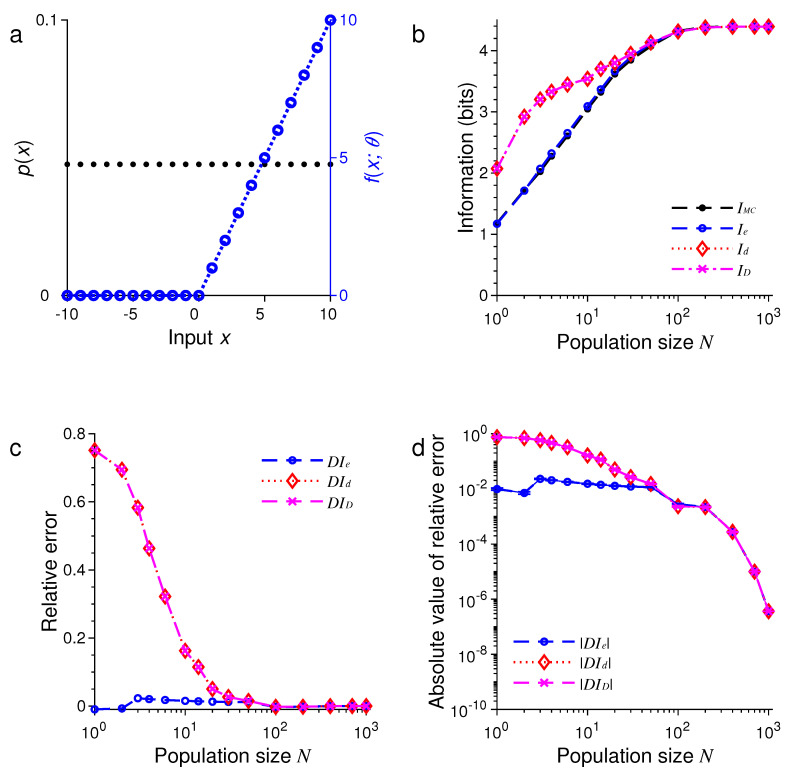
A comparison of approximations $I_{e}$, $I_{d}$ and $I_{D}$ against $I_{MC}$. The situation is identical to that in Figure 1 except for the shape of the tuning function (blue dashed lines in (**a**)). (**a**) Discrete uniform distribution of the stimulus p(x) (black dots) and the rectified linear tuning function $f\left(x;\;\theta \right)$ with center θ=0 (blue dashed lines); (**b**) The values of $I_{MC}$, $I_{e}$, $I_{d}$ and $I_{D}$ depend on the population size or total number of neurons *N*; (**c**) The relative errors $DI_{e}$, $DI_{d}$ and $DI_{D}$ for the results in (**b**); (**d**) The absolute values of the relative errors $|DI_{e}|$, $|DI_{d}|$ and $|DI_{D}|$ as in (**c**), with error bars showing standard deviations of repeated trials.

**Figure 4:**
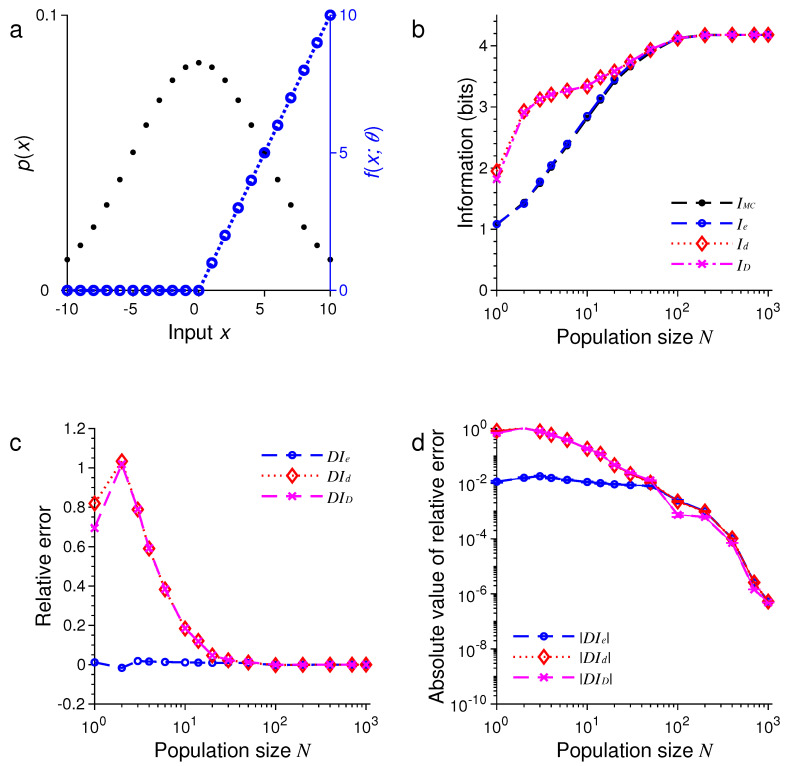
A comparison of approximations $I_{e}$, $I_{d}$ and $I_{D}$ against $I_{MC}$. The situation is identical to that in Figure 3 except that the stimulus distribution p(x) is peaked rather flat (black dots in (**a**)). (**a**) Discrete Gaussian-like distribution of the stimulus p(x) (black dots) and the rectified linear tuning function $f\left(x;\;\theta \right)$ with center θ=0 (blue dashed lines); (**b**) The values of $I_{MC}$, $I_{e}$, $I_{d}$ and $I_{D}$ depend on the population size or total number of neurons *N*; (**c**) The relative errors $DI_{e}$, $DI_{d}$ and $DI_{D}$ for the results in (**b**); (**d**) The absolute values of the relative errors $|DI_{e}|$, $|DI_{d}|$ and $|DI_{D}|$ as in (**c**), with error bars showing standard deviations of repeated trials.

Figure 3 and Figure 4 show the results under the same conditions of Figure 1 and Figure 2 except for the shape of the tuning functions.

**Figure 5:**
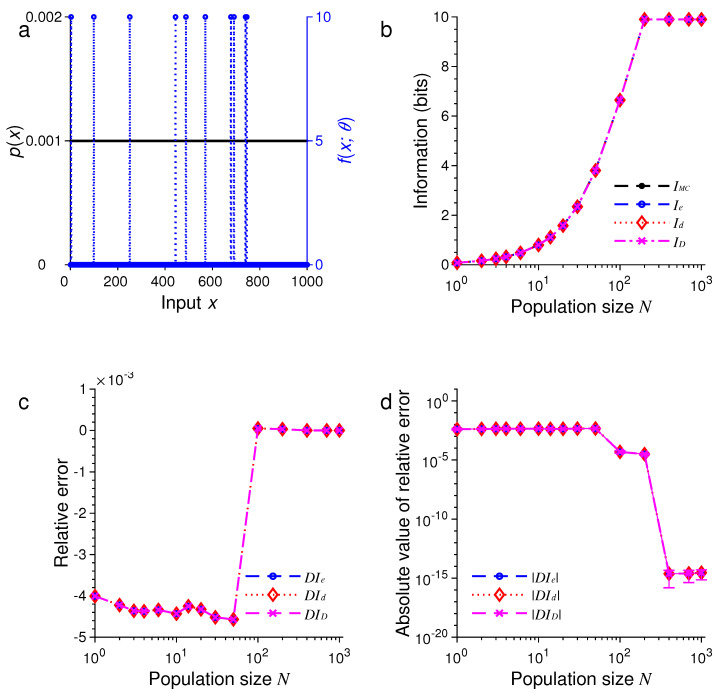
A comparison of approximations $I_{e}$, $I_{d}$ and $I_{D}$ against $I_{MC}$. The situation is similar to that in Figure 1 except that the tuning function is random (blue dashed lines in (**a**)); see Equation (66). (**a**) Discrete uniform distribution of the stimulus p(x) (black dots) and the random tuning function $f\left(x;\;\theta \right)$; (**b**) The values of $I_{MC}$, $I_{e}$, $I_{d}$ and $I_{D}$ depend on the population size or total number of neurons *N*; (**c**) The relative errors $DI_{e}$, $DI_{d}$ and $DI_{D}$ for the results in (**b**); (**d**) The absolute values of the relative errors $|DI_{e}|$, $|DI_{d}|$ and $|DI_{D}|$ as in (**c**), with error bars showing standard deviations of repeated trials.

**Figure 6:**
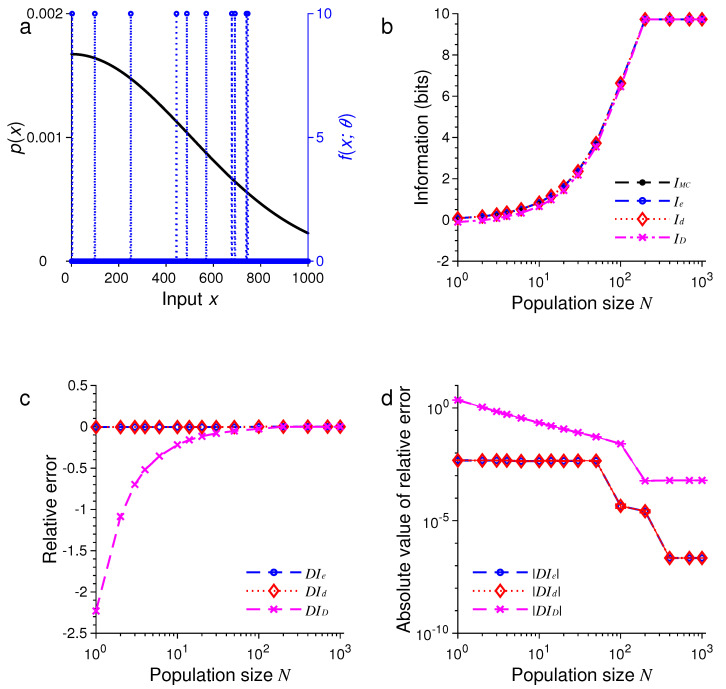
A comparison of approximations $I_{e}$, $I_{d}$ and $I_{D}$ against $I_{MC}$. The situation is identical to that in Figure 5 except that the stimulus distribution p(x) is not flat (black dots in (**a**)). (**a**) Discrete Gaussian-like distribution of the stimulus p(x) (black dots) and the random tuning function $f\left(x;\;\theta \right)$; (**b**) The values of $I_{MC}$, $I_{e}$, $I_{d}$ and $I_{D}$ depend on the population size or total number of neurons *N*; (**c**) The relative errors $DI_{e}$, $DI_{d}$ and $DI_{D}$ for the results in (**b**); (**d**) The absolute values of the relative errors $|DI_{e}|$, $|DI_{d}|$ and $|DI_{D}|$ as in (**c**), with error bars showing standard deviations of repeated trials.

Finally, we let the tuning function $f\left(x;\;\theta_{n}\right)$ have a random form:

(66) $$f\left(x;\;\theta_{n}\right)=\left\{\begin{array}{cc} B, & \mathrm{if}\;x\in \theta_{n}=\left\{\theta_{n}^{1},\;\theta_{n}^{2},\;\cdots ,\;\theta_{n}^{K}\right\},\\ 0, & \mathrm{otherwise}, \end{array}\right.$$

where the stimulus x∈X={1,2,⋯999,1000}, B=10, the values of $\left\{\theta_{n}^{1},\;\theta_{n}^{2},\;\cdots ,\;\theta_{n}^{K}\right\}$ are distinct and randomly selected from the set X with K=10. In this example, we may regard X as a list of natural objects (stimuli), and there are a total of *N* sensory neurons, each of which responds only to *K* randomly selected objects. Figure 5 shows the results under the condition that p(x) is a uniform distribution. In Figure 6, we assume that p(x) is not flat but a half Gaussian given by Equation (64) with σ=500.

In all these examples, we found that the three formulas, namely, $I_{e}$, $I_{d}$ and $I_{D}$, provided excellent approximations to the true values of mutual information as evaluated by Monte Carlo method. For example, in the examples in Figure 1 and Figure 5, all three approximations were practically indistinguishable. In general, all these approximations were extremely accurate when N>100.

In all our simulations, the mutual information tended to increase with the population size *N*, eventually reaching a plateau for large enough *N*. The saturation of information for large *N* is due to the fact that it requires at most $\log_{2}M$ bits of information to completely distinguish all *M* stimuli. It is impossible to gain more information than this maximum amount regardless of how many neurons are used in the population. In Figure 1, for instance, this maximum is $\log_{2}21=4.39$ bits, and in Figure 5, this maximum is $\log_{2}1000=9.97$ bits.

For relatively small values of *N*, we found that $I_{D}$ tended to be less accurate than $I_{e}$ or $I_{d}$ (see Figure 5 and Figure 6). Our simulations also confirmed two analytical results. The first one is that $I_{d}=I_{D}$ when the stimulus distribution is uniform; this result follows directly from the definitions of $I_{d}$ and $I_{D}$ and is confirmed by the simulations in Figure 1, Figure 3, and Figure 5. The second result is that $I_{d}=I_{e}$ (Equation (56)) when the tuning function is binary, as confirmed by the simulations in Figure 1, Figure 2, Figure 5, and Figure 6. When the tuning function allows many different values, $I_{e}$ can be much more accurate than $I_{d}$ and $I_{D}$, as shown by the simulations in Figure 3 and Figure 4. To summarize, our best approximation formula is $I_{e}$ because it is more accurate than $I_{d}$ and $I_{D}$, and, unlike $I_{d}$ and $I_{D}$, it applies to both discrete and continuous stimuli (Equations (10) and (13)).

## 4. Discussion

We have derived several asymptotic bounds and effective approximations of mutual information for discrete variables and established several relationships among different approximations. Our final approximation formulas involve only Kullback-Leibler divergence, which is often easier to evaluate than Shannon mutual information in practical applications. Although in this paper our theory is developed in the framework of neural population coding with concrete examples, our mathematical results are generic and should hold true in many related situations beyond the original context.

We propose to approximate the mutual information with several asymptotic formulas, including $I_{e}$ in Equation (10) or Equation (13), $I_{d}$ in Equation (15) and $I_{D}$ in Equation (18). Our numerical experimental results show that the three approximations $I_{e}$, $I_{d}$ and $I_{D}$ were very accurate for large population size *N*, and sometimes even for relatively small *N*. Among the three approximations, $I_{D}$ tended to be the least accurate, although, as a special case of $I_{d}$, it is slightly easier to evaluate than $I_{d}$. For a comparison of $I_{e}$ and $I_{d}$, we note that $I_{e}$ is the universal formula, whereas $I_{d}$ is restricted only to discrete variables. The two formulas $I_{e}$ and $I_{d}$ become identical when the responses or the tuning functions have only two values. For more general tuning functions, the performance of $I_{e}$ was better than $I_{d}$ in our simulations.

As mentioned before, an advantage of of $I_{e}$ is that it works not only for discrete stimuli but also for continuous stimuli. Theoretically speaking, the formula for $I_{e}$ is well justified, and we have proven that it approaches the true mutual information *I* in the limit of large population. In our numerical simulations, the performance of $I_{e}$ was excellent and better than that of $I_{d}$ and $I_{D}$. Overall, $I_{e}$ is our most accurate and versatile approximation formula, although, in some cases, $I_{d}$ and $I_{D}$ are slightly more convenient to calculate.

The numerical examples considered in this paper were based on an independent population of neurons whose responses have Poisson statistics. Although such models are widely used, they are appropriate only if the neural responses can be well characterized by the spike counts within a fixed time window. To study the temporal patterns of spike trains, one has to consider more complicated models. Estimation of mutual information from neural spike trains is a difficult computational problem [25,26,27,28]. In future work, it would be interesting to apply the asymptotic formulas such as $I_{e}$ to spike trains with small time bins each containing either one spike or nothing. A potential advantage of the asymptotic formula is that it might help reduce the bias caused by small samples in the calculation of the response marginal distribution $p\left(r\right)=\sum_{x}p\left(r|x\right)p\left(x\right)$ or the response entropy H(R) because here one only needs to calculate the Kullback-Leibler divergence $D\left(x||\hat{x}\right)$, which may have a smaller estimation error.

Finding effective approximation methods for computing mutual information is a key step for many practical applications of the information theory. Generally speaking, Kullback-Leibler divergence (Equation (7)) is often easier to evaluate and approximate than either Chernoff information (Equation (44)) or Shannon mutual information (Equation (1)). In situations where this is indeed the case, our approximation formulas are potentially useful. Besides applications in numerical simulations, the availability of a set of approximation formulas may also provide helpful theoretical tools in future analytical studies of information coding and representations.

As mentioned in the Introduction, various methods have been proposed to approximate the mutual information [9,10,11,12,13,14,15,16]. In future work, it would be useful to compare different methods rigorously under identical conditions in order to asses their relative merits. The approximation formulas developed in this paper are relatively easy to compute for practical problems. They are especially suitable for analytical purposes; for example, they could be used explicitly as objective functions for optimization or learning algorithms. Although the examples used in our simulations in this paper are parametric, it should be possible to extend the formulas to nonparametric problem, possibly with help of the copula method to take advantage of its robustness in nonparametric estimations [16]. 

## Appendix A. The Proofs

### Appendix A.1. Proof of Theorem 1

By Jensen’s inequality, we have

(A1) $$\begin{array}{cc} I_{\beta ,\alpha } & =-{\langle \ln\left(\int_{X}{\langle \frac{p^{\beta }\left(r|\hat{x}\right)p^{\alpha }\left(\hat{x}\right)}{p^{\beta }\left(r|x\right)p^{\alpha }\left(x\right)}\rangle }_{r|x}d\hat{x}\right)\rangle }_{x}+H\left(X\right)\\ & \leq -{\langle {\langle \ln\left(\int_{X}\frac{p^{\beta }\left(r|\hat{x}\right)p^{\alpha }\left(\hat{x}\right)}{p^{\beta }\left(r|x\right)p^{\alpha }\left(x\right)}d\hat{x}\right)\rangle }_{r|x}\rangle }_{x}+H\left(X\right) \end{array}$$

and

(A2) $$\begin{array}{c} -{\langle {\langle \ln\left(\int_{X}\frac{p^{\beta }\left(r|\hat{x}\right)p^{\alpha }\left(\hat{x}\right)}{p^{\beta }\left(r|x\right)p^{\alpha }\left(x\right)}d\hat{x}\right)\rangle }_{r|x}\rangle }_{x}+H\left(X\right)-I\\ ={\langle {\langle \ln\left(\int_{X}\frac{p(r,\hat{x})}{p\left(r,x\right)}d\hat{x}\right){\left(\int_{X}\frac{p^{\beta }\left(r|\hat{x}\right)p^{\alpha }\left(\hat{x}\right)}{p^{\beta }\left(r|x\right)p^{\alpha }\left(x\right)}d\hat{x}\right)}^{-1}\rangle }_{r|x}\rangle }_{x}\\ \leq \ln\int_{R}p\left(r\right)\frac{\int_{X}p^{\beta }\left(r|x\right)p^{\alpha }\left(x\right)dx}{\int_{X}p^{\beta }\left(r|\hat{x}\right)p^{\alpha }\left(\hat{x}\right)d\hat{x}}dr\\ =0. \end{array}$$

Combining Equations (A1) and (A2), we immediately get the lower bound in Equation (25).

In this section, we use integral for variable x, although our argument is valid for both continuous variables and discrete variables. For discrete variables, we just need to replace each integral by a summation, and our argument remains valid without other modification. The same is true for the response variable r.

To prove the upper bound, let

(A3) $$\Phi \left[q(\hat{x})\right]=\int_{R}p\left(r|x\right)\int_{X}q\left(\hat{x}\right)\ln\left(\frac{p\left(r|x\right)q\left(\hat{x}\right)}{p\left(r|\hat{x}\right)p\left(\hat{x}\right)}\right)d\hat{x}dr,$$

where $q(\hat{x})$ satisfies

(A4) $$\left\{\begin{array}{c} \int_{X}q\left(\hat{x}\right)d\hat{x}=1\\ q(\hat{x})\geq 0 \end{array}\right..$$

By Jensen’s inequality, we get

(A5) $$\Phi \left[q(\hat{x})\right]\geq \int_{R}p\left(r|x\right)\ln\left(\frac{p\left(r|x\right)}{p\left(r\right)}\right)dr.$$

To find a function $q(\hat{x})$ that minimizes $\Phi \left[q(\hat{x})\right]$, we apply the variational principle as follows:

(A6) $$\frac{\partial \tilde{\Phi }\left[q(\hat{x})\right]}{\partial q(\hat{x})}=\int_{R}p\left(r|x\right)\ln\left(\frac{p\left(r|x\right)q\left(\hat{x}\right)}{p\left(r|\hat{x}\right)p\left(\hat{x}\right)}\right)dr+1+\lambda ,$$

where λ is the Lagrange multiplier and

(A7) $$\tilde{\Phi }\left[q(\hat{x})\right]=\Phi \left[q(\hat{x})\right]+\lambda \left(\int_{X}q\left(\hat{x}\right)d\hat{x}-1\right).$$

Setting $\frac{\partial \tilde{\Phi }\left[q(\hat{x})\right]}{\partial q(\hat{x})}=0$ and using the constraint in Equation (A4), we find the optimal solution

(A8) $$q^{*}\left(\hat{x}\right)=\frac{p\left(\hat{x}\right)\exp\left(-D\left(x||\hat{x}\right)\right)}{\int_{X}p\left(\overset{ˇ}{x}\right)\exp\left(-D\left(x||\overset{ˇ}{x}\right)\right)d\overset{ˇ}{x}}.$$

Thus, the variational lower bound of $\Phi \left[q(\hat{x})\right]$ is given by

(A9) $$\Phi \left[q^{*}\left(\hat{x}\right)\right]=\min_{q(\hat{x})}\;\Phi \left[q(\hat{x})\right]=-\ln\left(\int_{X}p\left(\hat{x}\right)\exp\left(-D\left(x||\hat{x}\right)\right)d\hat{x}\right)dx.$$

Therefore, from Equations (1), (A5) and (A9), we get the upper bound in Equation (25). This completes the proof of Theorem 1.

### Appendix A.2. Proof of Theorem 2

It follows from Equation (43) that

(A10) $$\begin{array}{cc} I_{\beta_{1},\alpha_{1}} & =-{\langle \ln{\langle \exp\left(-\beta_{1}D_{\beta_{1}}\left(x||\hat{x}\right)+\left(1-\alpha_{1}\right)\ln\frac{p\left(x\right)}{p\left(\hat{x}\right)}\right)\rangle }_{\hat{x}}\rangle }_{x}\\ & \leq -{\langle \ln{\langle \exp\left(-e^{-1}D\left(x||\hat{x}\right)\right)\rangle }_{\hat{x}}\rangle }_{x}=I_{e}\\ & \leq -{\langle \ln{\langle \exp\left(-D\left(x||\hat{x}\right)\right)\rangle }_{\hat{x}}\rangle }_{x}=I_{u}, \end{array}$$

where $\beta_{1}=e^{-1}$ and $\alpha_{1}=1$. We immediately get Equation (26). This completes the proof of Theorem 2.

### Appendix A.3. Proof of Theorem 3

For the lower bound $I_{\beta ,\alpha }$, we have

(A11) $$\begin{array}{cc} I_{\beta ,\alpha } & =-\sum_{m=1}^{M}p\left(x_{m}\right)\ln\left(\sum_{\overset{ˇ}{m}=1}^{M}{\left(\frac{p\left(x_{\overset{ˇ}{m}}\right)}{p\left(x_{m}\right)}\right)}^{\alpha }\exp\left(-\beta D_{\beta }\left(x_{m}|x_{\overset{ˇ}{m}}\right)\right)\right)\\ & =-\sum_{m=1}^{M}p\left(x_{m}\right)\ln\left(1+d\left(x_{m}\right)\right)+H\left(X\right), \end{array}$$

where

(A12) $$d\left(x_{m}\right)=\sum_{\overset{ˇ}{m}\in M-\left\{m\right\}}{\left(\frac{p\left(x_{\overset{ˇ}{m}}\right)}{p\left(x_{m}\right)}\right)}^{\alpha }\exp\left(-\beta D_{\beta }\left(x_{m}|x_{{}_{\overset{ˇ}{m}}}\right)\right).$$

Now, consider

(A13) $$\begin{array}{c} \ln\left(1+d\left(x_{m}\right)\right)\\ =\ln\left(1+a\left(x_{m}\right)+b\left(x_{m}\right)\right)\\ =\ln\left(1+a\left(x_{m}\right)\right)+\ln\left(1+b\left(x_{m}\right){\left(1+a\left(x_{m}\right)\right)}^{-1}\right)\\ =\ln\left(1+a\left(x_{m}\right)\right)+O\left(N^{-\gamma }\right), \end{array}$$

where

(A14a) $$\begin{array}{cc} a\left(x_{m}\right) & =\sum_{\hat{m}\in M_{m}^{\beta }}{\left(\frac{p\left(x_{\hat{m}}\right)}{p\left(x_{m}\right)}\right)}^{\alpha }\exp\left(-\beta D_{\beta }\left(x_{m}\left|\right|x_{\hat{m}}\right)\right), \end{array}$$

(A14b) $$\begin{array}{cc} b\left(x_{m}\right) & =\sum_{\overset{ˇ}{m}\in {M-M}_{m}^{\beta }}{\left(\frac{p\left(x_{\overset{ˇ}{m}}\right)}{p\left(x_{m}\right)}\right)}^{\alpha }\exp\left(-\beta D_{\beta }\left(x_{m}\left|\right|x_{\overset{ˇ}{m}}\right)\right)\\ & \leq N^{-\gamma_{1}}\sum_{\overset{ˇ}{m}\in {M-M}_{m}^{\beta }}{\left(\frac{p\left(x_{\overset{ˇ}{m}}\right)}{p\left(x_{m}\right)}\right)}^{\alpha }=O\left(N^{-\gamma_{1}}\right). \end{array}$$

Combining Equations (A11) and (A13) and Theorem 1, we get the lower bound in Equation (30). In a manner similar to the above, we can get the upper bound in Equations (30) and (31). This completes the proof of Theorem 3.

### Appendix A.4. Proof of Theorem 4

The upper bound $I_{u}$ for mutual information *I* in Equation (25) can be written as

(A15) $$\begin{array}{cc} I_{u} & =-\int_{X}\left(\ln\int_{X}p\left(\hat{x}\right)\exp\left(-D\left(x|\hat{x}\right)\right)d\hat{x}\right)p\left(x\right)dx\\ & =-{\langle \ln\left(\int_{X}\exp\left({\langle L\left(r|\hat{x}\right)-L\left(r|x\right)\rangle }_{r|x}\right)d\hat{x}\right)\rangle }_{x}+H\left(X\right). \end{array}$$

where $L\left(r|\hat{x}\right)=\ln\left(p\left(r|\hat{x}\right)p\left(\hat{x}\right)\right)$ and $L\left(r|x\right)=\ln\left(p\left(r|x\right)p\left(x\right)\right)$.

Consider the Taylor expansion for $L(r|\hat{x})$ around x. Assuming that $L(r|\hat{x})$ is twice continuously differentiable for any $\hat{x}\in X_{\omega }\left(x\right)$, we get

(A16) $$\begin{array}{c} {\langle L\left(r|\hat{x}\right)-L\left(r|x\right)\rangle }_{r|x}\\ ={y^{T}v}_{1}-\frac{1}{2}y^{T}y-\frac{1}{2}y^{T}G{}^{-1/2}\left(x\right)\left(G\left(\overset{˘}{x}\right)-G\left(x\right)\right)G{}^{-1/2}\left(x\right)y \end{array}$$

where

(A17) $$y=G{}^{1/2}\left(x\right)(\hat{x}-x),$$

(A18) $$v{}_{1}=G{}^{-1/2}\left(x\right)q^{\prime }\left(x\right)$$

and

(A19) $$\overset{˘}{x}=x+t\left(\hat{x}-x\right)\in X_{\omega }\left(x\right),\;t\in \left(0,1\right).$$

For later use, we also define

(A20) $$v=G{}^{-1/2}\left(x\right)l^{\prime }\left(r|x\right)$$

where

(A21) $$l\left(r|x\right)=\ln p\left(r|x\right).$$

Since $G(\overset{˘}{x})$ is continuous and symmetric for $\overset{˘}{x}\in X$, for any $\epsilon \in \left(0,\;1\right)$, there is a $\varepsilon \in \left(0,\;\omega \right)$ such that

(A22) $$\left|y^{T}G{}^{-1/2}\left(x\right)\left(G\left(\overset{˘}{x}\right)-G\left(x\right)\right)G{}^{-1/2}\left(x\right)y\right|<\epsilon {∥y∥}^{2}$$

for all $y\in Y_{\varepsilon }$, where $Y_{\varepsilon }=\left\{y\in R^{K}:∥y∥<\varepsilon \sqrt{N}\right\}$. Then, we get

(A23) $$\begin{array}{c} \ln\left(\int_{X}\exp\left({\langle L\left(r|\hat{x}\right)-L\left(r|x\right)\rangle }_{r|x}\right)d\hat{x}\right)\\ \geq -\frac{1}{2}\ln\left(\det\left(G(x)\right)\right)+\ln\int_{Y_{\varepsilon }}\exp\left({y^{T}v}_{1}-\frac{1}{2}\left(1+\epsilon \right)y^{T}y\right)dy \end{array}$$

and with Jensen’s inequality,

(A24) $$\begin{array}{c} \ln\int_{Y_{\varepsilon }}\exp\left({y^{T}v}_{1}-\frac{1}{2}\left(1+\epsilon \right)y^{T}y\right)dy\\ \geq \ln\Psi_{\varepsilon }+\int_{{\hat{Y}}_{\varepsilon }}{y^{T}v}_{1}\phi_{\varepsilon }\left(y\right)dy\\ =\frac{K}{2}\ln\left(\frac{2\pi }{1+\epsilon }\right)+O\left(N^{-K/2}e^{-N\delta }\right), \end{array}$$

where δ is a positive constant, $\int_{{\hat{Y}}_{\varepsilon }}{y^{T}v}_{1}\phi_{\varepsilon }\left(y\right)dy=0$,

(A25) $$\left\{\begin{array}{c} \phi_{\varepsilon }\left(y\right)=\Psi_{\varepsilon }^{-1}\exp\left(-\frac{1}{2}\left(1+\epsilon \right)y^{T}y\right)\\ \Psi_{\varepsilon }=\int_{{\hat{Y}}_{\varepsilon }}\exp\left(-\frac{1}{2}\left(1+\epsilon \right)y^{T}y\right)dy \end{array}\right.$$

and

(A26) $${\hat{Y}}_{\varepsilon }=\left\{y\in R^{K}:\left|y_{k}\right|<\varepsilon \sqrt{N/K},\;k=1,2,\cdots ,K\right\}\subseteq Y_{\varepsilon }.$$

Now, we evaluate

(A27) $$\begin{array}{cc} \Psi_{\varepsilon } & =\int_{R^{K}}\exp\left(-\frac{1}{2}\left(1+\epsilon \right)y^{T}y\right)dy-\int_{R^{K}-{\hat{Y}}_{\varepsilon }}\exp\left(-\frac{1}{2}\left(1+\epsilon \right)y^{T}y\right)dy\\ & ={\left(\frac{2\pi }{1+\epsilon }\right)}^{K/2}-\int_{R^{K}-{\hat{Y}}_{\varepsilon }}\exp\left(-\frac{1}{2}\left(1+\epsilon \right)y^{T}y\right)dy. \end{array}$$

Performing integration by parts with $\int_{a}^{\infty }e^{-t^{2}/2}dt=\frac{e^{-a^{2}/2}}{a}-\int_{a}^{\infty }\frac{e^{-t^{2}/2}}{t^{2}}dt$, we find

(A28) $$\begin{array}{cc} \int_{R^{K}-{\hat{Y}}_{\varepsilon }}\exp\left(-\frac{1}{2}\left(1+\epsilon \right)y^{T}y\right)dy & \leq \frac{\exp\left(-\frac{1}{2}\left(1+\epsilon \right)\varepsilon^{2}N\right)}{{\left({\left(1+\epsilon \right)}^{2}\varepsilon^{2}N/\left(4K\right)\right)}^{K/2}}\\ & =O\left(N^{-K/2}e^{-N\delta }\right), \end{array}$$

for some constant δ>0.

Combining Equations (A15), (A23) and (A24), we get

(A29) $$I_{u}\leq \frac{1}{2}{\langle \ln\left(\det\left(\frac{\left(1+\epsilon \right)}{2\pi }G\left(x\right)\right)\right)\rangle }_{x}+H\left(X\right)+O\left(N^{-K/2}e^{-N\delta }\right).$$

On the other hand, from Equation (A22) and the condition in Equation (33), we obtain

(A30) $$\begin{array}{c} \int_{X_{\varepsilon }\left(x\right)}\exp\left({\langle L\left(r|\hat{x}\right)-L\left(r|x\right)\rangle }_{r|x}\right)d\hat{x}\\ \leq \det{\left(G(x)\right)}^{-1/2}\int_{R^{K}}\exp\left({y^{T}v}_{1}-\frac{1}{2}\left(1-\epsilon \right)y^{T}y\right)dy\\ =\det{\left(\frac{1-\epsilon }{2\pi }G\left(x\right)\right)}^{-1/2}\exp\left(\frac{1}{2}{\left(1-\epsilon \right)}^{-1}v^{T}v_{1}\right) \end{array}$$

and

(A31) $$\begin{array}{c} \int_{X}\exp\left({\langle L\left(r|\hat{x}\right)-L\left(r|x\right)\rangle }_{r|x}\right)d\hat{x}\\ =\int_{X_{\varepsilon }\left(x\right)}\exp\left({\langle L\left(r|\hat{x}\right)-L\left(r|x\right)\rangle }_{r|x}\right)d\hat{x}+\int_{{X-X}_{\varepsilon }\left(x\right)}\exp\left({\langle L\left(r|\hat{x}\right)-L\left(r|x\right)\rangle }_{r|x}\right)d\hat{x}\\ \leq \det{\left(\frac{1-\epsilon }{2\pi }G\left(x\right)\right)}^{-1/2}\left(\exp\left(\frac{v^{T}v_{1}}{2\left(1-\epsilon \right)}\right)+O\left(N^{-1}\right)\right). \end{array}$$

It follows from Equations (A15) and (A31) that

(A32) $$\begin{array}{c} {\langle \ln\left(\int_{X}\exp\left({\langle L\left(r|\hat{x}\right)-L\left(r|x\right)\rangle }_{r|x}\right)d\hat{x}\right)\rangle }_{x}\\ \leq -\frac{1}{2}{\langle \ln\left(\det\left(\frac{\left(1-\epsilon \right)}{2\pi }G\left(x\right)\right)\right)\rangle }_{x}+\frac{1}{2}{\left(1-\epsilon \right)}^{-1}{\langle v^{T}v_{1}\rangle }_{x}+O\left(N^{-1}\right). \end{array}$$

Note that

(A33) $${\langle v^{T}v_{1}\rangle }_{x}=O\left(N^{-1}\right).$$

Now, we have

(A34) $$I_{u}\geq \frac{1}{2}{\langle \ln\left(\det\left(\frac{\left(1-\epsilon \right)}{2\pi }G\left(x\right)\right)\right)\rangle }_{x}+H\left(X\right)+O\left(N^{-1}\right).$$

Since ϵ is arbitrary, we can let it go to zero. Therefore, from Equations (25), (A29) and (A34), we obtain the upper bound in Equation (35).

The Taylor expansion of $h\left(\hat{x},x\right)={\langle {\left(\frac{p(r|\hat{x})}{p\left(r|x\right)}\right)}^{\beta }\rangle }_{r|x}$ around x is

(A35) $$\begin{array}{cc} h\left(\hat{x},x\right) & =1+{\langle \frac{\beta }{p(r|x)}\frac{\partial p(r|x)}{\partial x}\rangle }_{r|x}(\hat{x}-x)+\\ & (\hat{x}-{x)}^{T}{\langle \frac{\beta }{2p{\left(r|x\right)}^{2}}\left(\left(\beta -1\right)\frac{\partial p(r|x)}{\partial x}\frac{\partial p(r|x)}{\partial x^{T}}+p\left(r|x\right)\frac{\partial^{2}p\left(r|x\right)}{\partial x\partial x^{T}}\right)\rangle }_{r|x}(\hat{x}-x)+\cdots \\ & =1-\frac{\beta \left(1-\beta \right)}{2}(\hat{x}-{x)}^{T}J\left(x\right)(\hat{x}-x)+\cdots . \end{array}$$

In a similar manner as described above, we obtain the asymptotic relationship (37):

(A36) $$\begin{array}{cc} I_{\beta ,\alpha } & =I_{\gamma }+O\left(N^{-1}\right)\\ & =\frac{1}{2}\int_{X}p\left(x\right)\ln\left(\det\left(\frac{G_{\gamma }\left(x\right)}{2\pi }\right)\right)dx+H\left(X\right). \end{array}$$

Notice that $0<\gamma =\beta \left(1-\beta \right)\leq 1/4$ and the equality holds when $\beta =\beta_{0}=1/2$. Thus, we have

(A37) $$\det\left(G_{\gamma }\left(x\right)\right)\leq \det\left(G_{\gamma_{0}}\left(x\right)\right).$$

Combining Equations (25), (A36) and (A37) yields the lower bound in Equation (35).

The proof of Equation (36) is similar. This completes the proof of Theorem 4.
